# Non-mercaptalbumin is significantly associated with the coronary plaque burden and the severity of coronary artery disease

**DOI:** 10.1038/s41598-021-93753-0

**Published:** 2021-07-09

**Authors:** Shengpu Chou, Keiko Yasukawa, Yusuke Fujino, Midori Ishibashi, Mikiko Haraguchi, Masaya Sato, Hitoshi Ikeda, Sunao Nakamura, Yutaka Yatomi

**Affiliations:** 1 Cardiovascular Department, New Tokyo Hospital, 1271 Wanagaya, Matsudo, Chiba 270-2232 Japan; 2 Diabetes and Metabolic Department, New Tokyo Hospital, 1271 Wanagaya, Matsudo, Chiba 270-2232 Japan; 3 Department of Clinical Laboratory, The University of Tokyo Hospital, 7-3-1 Hongo, Bunkyo-Ku, Tokyo, 113-8655 Japan; 4 Cardiovascular Department, Kashiwa Kousei General Hospital, 617 Shikoda, Kashiwa, Chiba 277-8551 Japan; 5 Department of Clinical Laboratory Medicine, New Tokyo Hospital, 1271 Wanagaya, Matsudo, Chiba 270-2232 Japan

**Keywords:** Biomarkers, Cardiology, Medical research

## Abstract

Human non-mercaptalbumin (HNA), oxidized form of serum albumin, has been reported as a useful marker in oxidative stress-related diseases; however, few reports have examined the association between HNA and the severity of coronary artery disease (CAD). The present study evaluated whether the HNA fraction is correlated with coronary artery stenosis in 140 patients considered to have a high risk of CAD or who were suspected of having acute coronary syndrome. The severity of CAD was defined by the number of stenotic coronary vessels and a severity score system (the Gensini score). HNA measurements were performed using our newly established high-performance liquid chromatography methodology. The results had shown that HNA was significantly increased in patients with three-vessel disease, compared with those without CAD or with single-vessel disease (*p* = 0.025), and was positively correlated with the Gensini score (ρ = 0.421, *p* < 0.001). A multivariate analysis showed that the number of stenotic vessels was an independent and significant factor associated with HNA (ρ = 1.246, *p* = 0.012). A logistic regression analysis showed that HNA was a strong predictor of multivessel CAD (odds ratio, 1.12; 95% confidence interval, 1.020–1.229; *p* = 0.017). These findings indicate that the measurement of HNA could be clinically practical for predicting the severity of coronary artery stenosis.

## Introduction

Coronary artery disease (CAD) remains the leading cause of death in Europe and the United States^1^. For decades, atherosclerosis has been believed to be the core mechanism of undesirable plaque formation in coronary vessels^2^. While many conventional coronary risk factors such as genetic disposition, diabetes, hypertension, renal dysfunction, smoking and dyslipidemia have been shown to predict the progression of atherosclerotic disorders, there continues to be a need for new markers that allow early prediction and intervention of coronary stenosis.

Atherosclerosis develops through chronic inflammatory cellular responses that lead to ectopic calcification and lipid micro-deposits on damaged arterial endothelium; oxidative and anti-oxidative stress imbalances also seem to contribute to the endothelial inflammatory response and plaque formation^2–5^. Since excess reactive oxygen species (ROS) may accelerate atherogenic plaque formation, the extent of oxidative stress in the systemic circulation could be both a reasonable and effective means of predicting the extent of atherosclerotic changes^6^*.*

Serum albumin is the most abundant protein in human blood plasma; its functions, such as electrolyte buffering, the regulation of thyroid hormones, the determination of oncotic pressure, and the metabolism of various pharmaceuticals, has been widely studied^7,8^. Additionally, albumin is thought to regulate the redox state of the systemic circulation because albumin exists as oxidized human non-mercaptalbumin (HNA) and reduced human mercaptalbumin (HMA). The free SH group of Cys34 of HMA captures superoxide or free hydroxyl radical and is converted to HNA^9,10^. A recently developed method for measuring HNA and HMA using high-performance liquid chromatography (HPLC) has improved measurement accuracy and speed^11,12^.

HNA was previously reported to be correlated with chronic liver disease, renal dysfunction, and various diabetic complications^13–18^. However, there are few reports on the clinical relationship between the redox status of serum albumin and the severity of CAD and acute coronary syndrome (ACS). The present study therefore aimed to evaluate the correlation between HNA and the severity of CAD (defined by the number of stenotic vessels and the Gensini score) in patients with a high risk of CAD or who were suspected of having ACS^19^.

## Results

### Patient demographics and clinical parameters

Among the 140 patients enrolled in this study, two patients were excluded from the analysis. One patient withdrew consent, and the second was excluded because of a diagnosis of cardiac sarcoidosis, which was thought to make him unsuitable for inclusion in the analysis. The average HNA was 26.4% ± 5.35%. The average age was 66.9 years, and 71% of the patients were male. The extent of vessel disease was determined as 0, 1, 2, and 3 vessels in 35, 40, 36, and 27 patients, respectively. Eighty-two patients (59%) were diagnosed as having ACS. Thirty-five subjects were current smokers (25%), and 55 had been previously diagnosed as having diabetes (40%). The average initial CPK, CK-MB, and troponin I values were 885 IU/L, 33.0 IU/L, and 4290 pg/mL, respectively. The average eGFR was 66.7 mL/min/1.73 m^2^. All other clinical parameters and blood examinations are summarized in Table 1.

**Table 1:** Baseline patient clinical characteristics (n = 138).

Characteristic	Value	Blood examination	Value
Age (year)	66.9 ± 13.2	HNA%	26.4 ± 5.35
BMI (kg/m^2^)	24.8 ± 5.1	BUN (mg/dL)	16.0 ± 5.2
Male/female	98/40 (71/29)	Cre (mg/dL)	1.05 ± 0.48
No. of involved vessels (0/1/2/3)	35/40/36/27 (25/29/26/20)	eGFR (mL/min/1.73 m^2^)	66.7 ± 18.5
Multivessel CAD (yes/no)	63/75 (46/64)	HbA1c (%)	6.57 ± 1.36
ACS (yes/no)	82/56 (59/41)	Hb (g/dL)	13.8 ± 1.85
Current smoker (yes/no)	35/103 (25/75)	WBC (/µL)	8460 ± 3960
Dyslipidemia (yes/no)	94/44 (68/32)	PLT (10^4/^μL)	21.9 ± 6.50
SBP > 140 mmHg (yes/no)	39/99 (28/72)	hs-CRP (mg/dL)	0.85 ± 2.14
DM (yes/no﻿)	55/83 (40/60)	CPK (IU/L)	885 ± 1680
		CK-MB (IU/L)	33.0 ± 96.9
		TnI (pg/mL)	4290 ± 17,940
		BNP (pg/mL)	132 ± 400
		TP (g/dL)	7.08 ± 0.85
		Alb (g/dL)	3.96 ± 0.44
		HDL-C (mg/dL)	49.1 ± 12.5
		LDL-C (mg/dL)	119.7 ± 42.1
		TG (mg/dL)	166.9 ± 144.2

The numbers are reported as number (%) or average ± standard deviation, unless otherwise described. Details regarding the abbreviations can be found in the “List of abbreviations” section.

### Associations between HNA and angiographic severity of CAD

**Figure 1:**
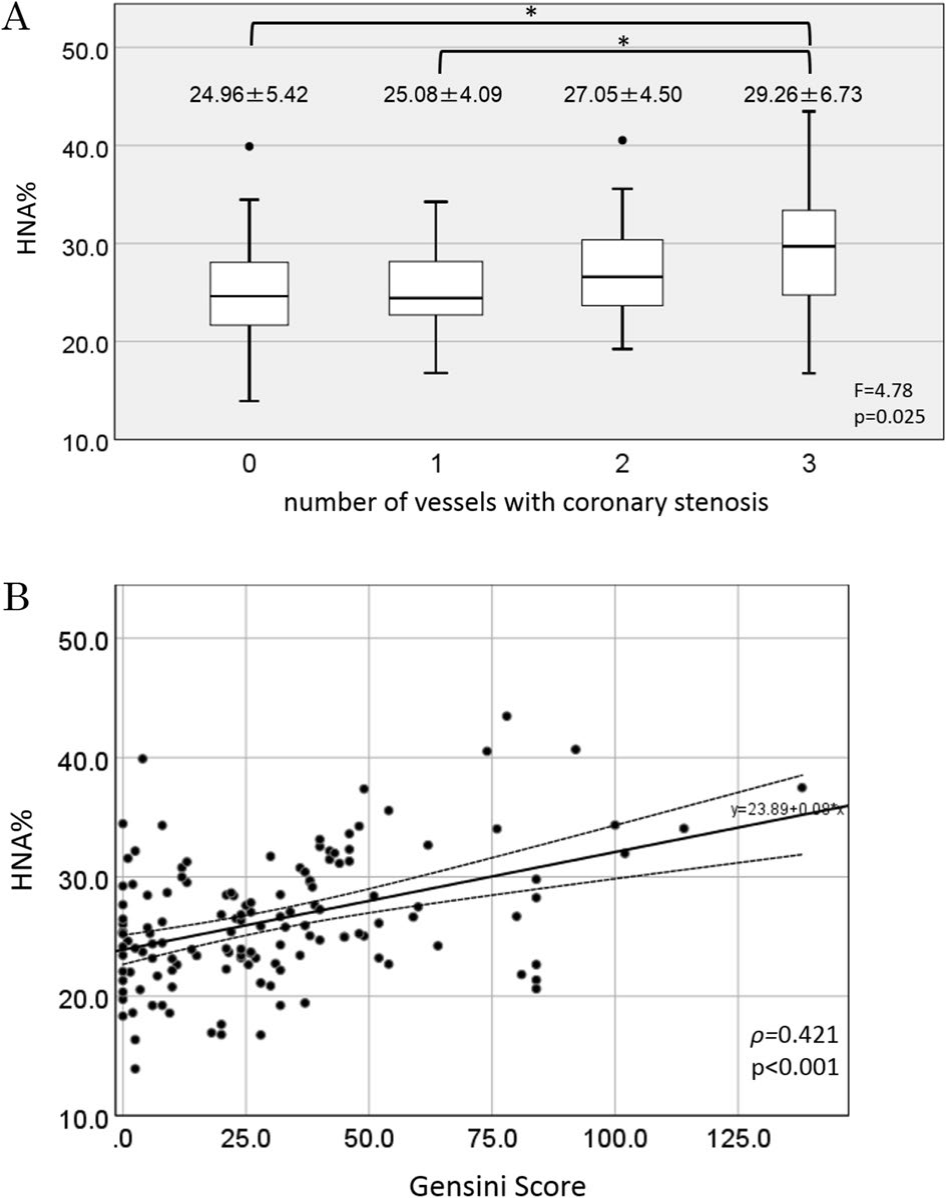
Associations between HNA and the angiographic severity of CAD as evaluated by the stenotic vessels (**A**) and the Gensini score (**B**). In (**A**), the reported numbers are the average ± standard deviation. The box shows the 1st–3rd quartiles; the bold line shows the 2nd quartiles; the whiskers show the 95th minimum and maximum values; the filled circles are outliers. The width of the box shows the statistical degrees of freedom. The *p* value is for a 4-group comparison using a one-way analysis of variance. In (**B**), the bold and dotted lines represent linear regression and the 95% confidence interval, respectively.

Significant differences were observed between the HNA level and the number of stenotic coronary artery vessels, with significantly higher HNA values observed in patients with more extensive vessel coronary disease (24.96% ± 5.42%, 25.08% ± 4.09%, 27.05% ± 4.50%, and 29.29% ± 6.73% for 0-, 1-, 2-, and 3-vessel disease, respectively). This relationship was weak, but significant (ANOVA F = 4.78, *p* = 0.025). Further post-hoc comparison of the Bonferroni corrections showed that patients with three-vessel disease had a significantly higher HNA level than those without coronary stenosis or those with single-vessel disease (Fig. 1A). Moreover, the HNA level was positively correlated with the degree of coronary stenosis as evaluated using the Gensini score (ρ = 0.421, *p* < 0.001) (Fig. 1B).

### Correlations between clinical parameters and HNA

Next, we performed a Pearson correlation analysis to investigate the correlations between the patients’ clinical parameters and the HNA level. The analyses showed that the extent of vessel disease, patient age, and BUN, creatinine, troponin I, BNP, and hs-CRP levels were significantly and positively correlated with the HNA level (*p* < 0.001 except for hs-CRP, which was *p* < 0.005). Furthermore, albumin was significantly and negatively correlated with the HNA level (*p* < 0.001). The details and the Pearson correlations (ρ) are shown in Table 2.

**Table 2:** Bivariate analysis of clinical parameters and HNA.

	ρ	*p*		ρ	*p*
Age	0.345	< 0.001*	TP	− 0.069	0.430
Vessel disease	0.292	< 0.001*	Alb	− 0.294	< 0.001*
CPK	0.063	0.464	BUN	0.397	< 0.001*
CK-MB	0.105	0.262	Cre	0.420	< 0.001*
TnI	0.311	< 0.001*	WBC	0.111	0.193
BNP	0.333	< 0.001*	Hb	− 0.148	0.083
Smoking	− 0.077	0.368	PLT	− 0.034	0.691
ACS	0.125	0.144	HbA1c	0.065	0.462
Male	− 0.024	0.777	DM	0.123	0.152
BMI	− 0.162	0.057	hs-CRP	0.278	0.003*
HT	0.059	0.495	LDL-C	− 0.011	0.895
UA	0.145	0.089	HDL-C	− 0.117	0.171
			TG	− 0.033	0.702

The extent of vessel disease as well as the patient age and troponin I, BNP, BUN, creatinine, and hs-CRP levels were positively correlated with HNA, while the serum albumin level showed a negative correlation with HNA. *Statistically significant as *p* < 0.05.

### Multivariate analysis of various factors to predict the value of HNA

According to the results shown in Table 2, not only the number of stenotic coronary vessels, age, and renal function, but also parameters such as the troponin I, BNP, and hs-CRP levels were correlated with the HNA level. Thus, we further investigated these parameters that were associated with the HNA level using multivariate analyses. Five models were proposed. The results showed that the extent of vessel disease was significantly and independently associated with HNA in all 5 models, whereas the hs-CRP, HbA1c, BMI, troponin I, and BNP levels were not significantly associated with the HNA level in multivariate models 1, 2, 3 and 4 (Table 3). These findings suggest that the variation in HNA is independent of existing myocardium-related parameters, and the measurement of these parameters at a single time point might not be useful for predicting the HNA level.

**Table 3:** Multivariate analyses of various myocardium-related factors associated with HNA.

	Model 1		Model 2		Model 3		Model 4		Model 5	
	ρ	*p*	ρ	*p*	ρ	*p*	ρ	*p*	ρ	*p*
Vessel disease	1.099	0.034	1.105	0.030	1.101	0.030	1.158	0.021	1.246	0.012
Age	0.081	0.084	0.079	0.068	0.092	0.019	0.087	0.024	0.080	0.035
Cre	3.078	0.006	3.057	0.003	3.320	0.001	3.358	0.001	3.570	0.001
hs-CRP	0.264	0.345	0.241	0.340	0.275	0.260	0.257	0.292	–	–
HbA1c	0.315	0.350	0.322	0.332	0.304	0.358	–	–	–	–
BMI	− 0.070	0.482	− 0.071	0.456	–	–	–	–	–	–
TnI	3.23E−5	0.550	3.019E−5	0.515	–	–	–	–	–	–
BNP	− 0.01	0.803	–	–	–	–	–	–	–	–
BUN	0.018	0.887	–	–	–	–	–	–	–	–
R^2^	0.319 (*p* < 0.001)		0.318 (*p* < 0.001)		0.310 (*p* < 0.001)		0.304 (*p* < 0.001)		0.296 (*p* < 0.001)	

All five models showed that the extent of vessel disease, patient age, and creatinine level were significantly independent factors associated with the HNA level. However, hs-CRP, HbA1c, BMI, troponin I, BNP, and BUN were not significantly associated with HNA. *R*^*2*^ multiple coefficients of determination.

### Associations between HNA and the diagnosis of ACS

**Figure 2:**
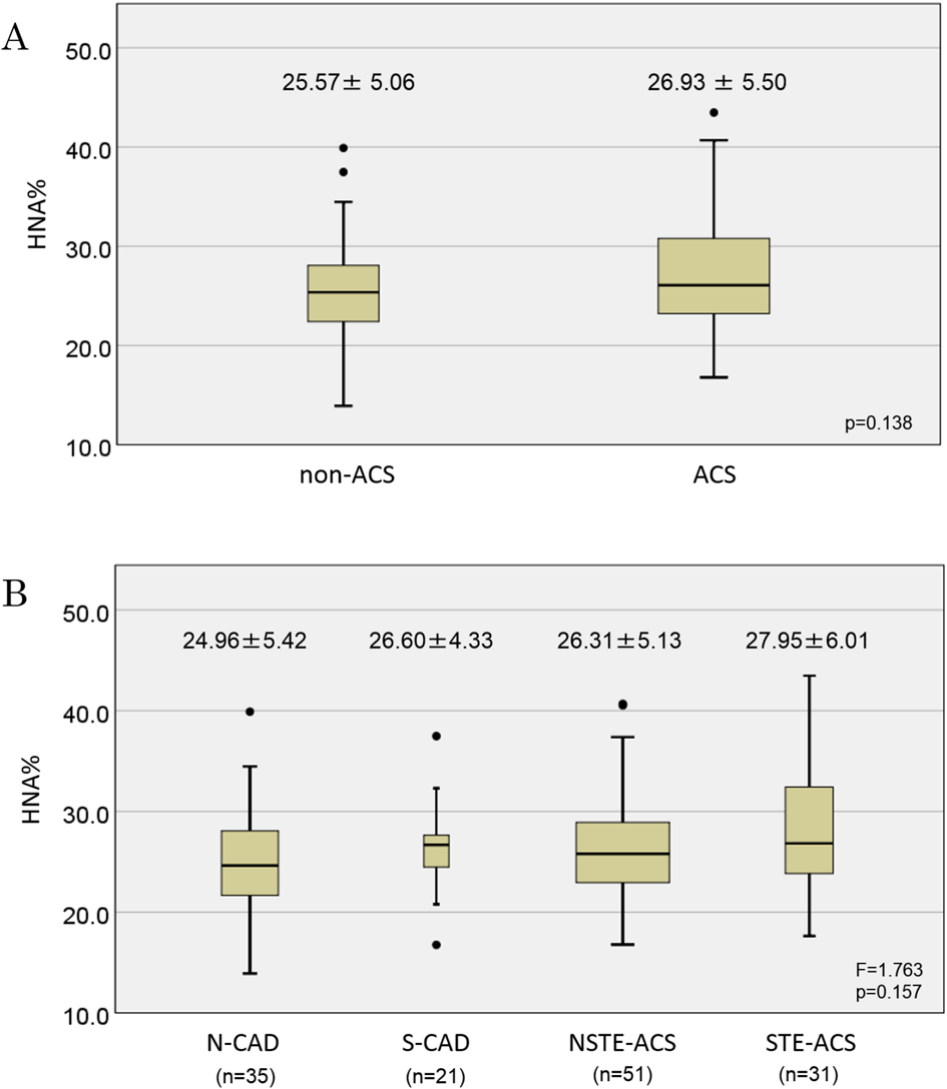
(**A**) Comparison of HNA in patients diagnosed with ACS (n = 82) and those without ACS (n = 56). (**B**) Comparisons of HNA among groups categorized according to the final diagnosis. N-CAD: without coronary artery disease; S-CAD: stable coronary artery disease; NSTE-ACS: non-ST elevation acute coronary syndrome; STE-ACS: ST elevation acute coronary syndrome.

We next examined the influences of ACS on HNA and attempted to identify a difference in HNA levels between subjects with and those without a diagnosis of ACS. The results indicated that HNA was not useful for supporting a diagnosis of ACS, since no significant difference in HNA levels was seen between non-ACS patients (including non-CAD and stable CAD) and patients with ACS (25.57% ± 5.06% vs. 26.93% ± 5.50%; *p* = 0.138). Moreover, detailed categorical diagnoses were determined after considering the clinical manifestations, blood examinations, and CAG or coronary CTA images, and no significant differences in HNA were seen among patients diagnosed as having non-CAD, stable CAD, non-ST elevation ACS (NSTE-ACS), or ST elevation ACS (STE-ACS) (24.96% ± 5.42%, 26.60% ± 4.33%, 26.31% ± 5.13%, and 27.95% ± 6.01%, respectively; F = 1.763, *p* = 0.157), indicating that emergent ACS, ST elevation or non-ST elevation myocardial damage did not affect the HNA value in a notable manner (Fig. 2A,B).

### Impact of diabetes mellitus on HNA in patients with and those without coronary artery stenosis

**Figure 3:**
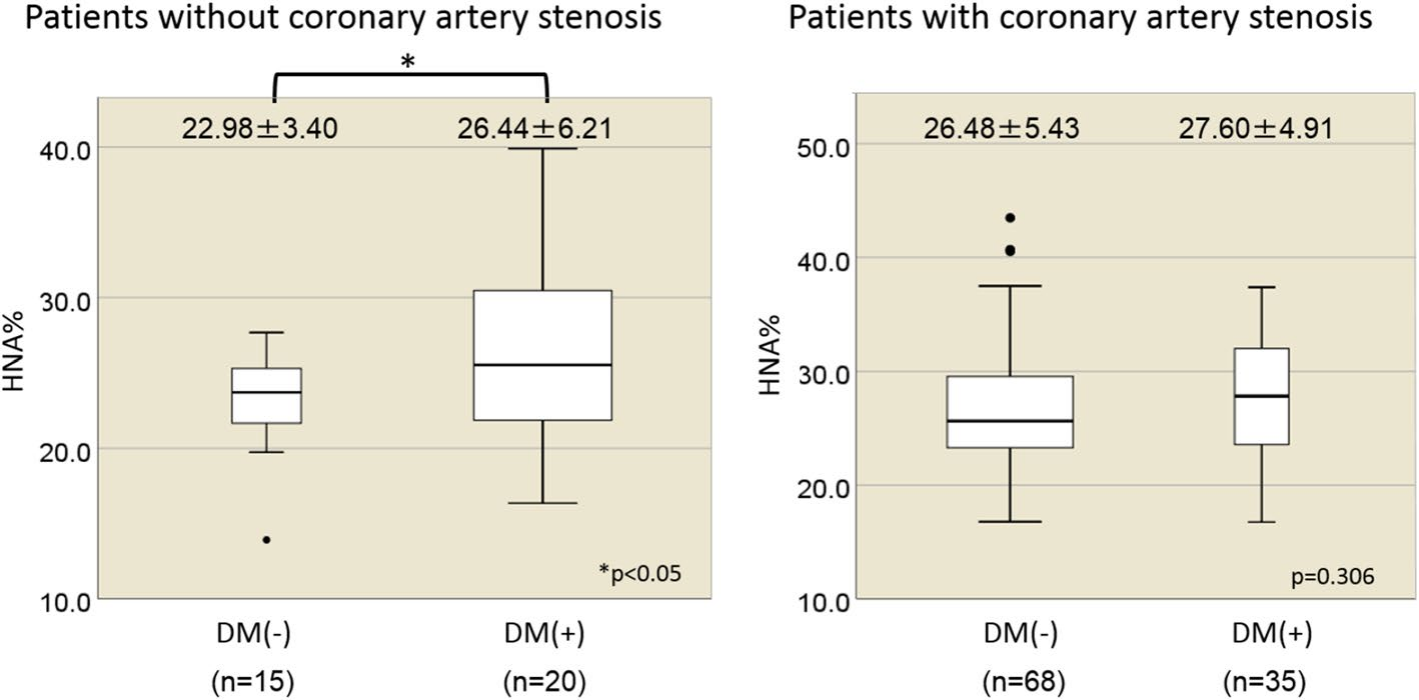
Impact of diabetes mellitus on HNA in patients with and those without coronary artery stenosis. Among patients without coronary artery stenosis, the HNA level was significantly higher in diabetes patients than in non-diabetic subjects. However, the correlation disappeared among patients who had significant coronary artery stenosis.

Diabetes is a well-known coronary risk factor. Although the HbA1c level was not correlated with the HNA level, we investigated the impact of diabetes on HNA in a detailed analysis. The results showed that among patients without significant coronary artery stenosis, HNA was significantly increased in subjects who had diabetes, compared with those without diabetes (26.44% ± 6.21% vs. 22.98% ± 3.40%; *p* = 0.044). However, in subjects who had been diagnosed with at least one vessel of coronary artery stenosis, no difference in HNA levels was seen between diabetic and non-diabetic patients (27.60% ± 4.91% vs. 26.48% ± 5.43%; *p* = 0.306) (Fig. 3).

### Logistic regression analysis to predict multivessel CAD

To evaluate whether the HNA level might be useful for the prediction of multivessel CAD, a logistic regression analysis was performed. An unadjusted analysis (Model 1) showed that a 1% increase in HNA was significantly associated with an 11.7% increase in the risk of multivessel CAD. Furthermore, age and the HDL-C level were also significant factors associated with multivessel CAD. The LDL-C level, systemic blood pressure, and total protein, troponin I, BUN, and HbA1c levels did not reach statistical significance.

In model 2, in which the troponin I, BUN, and HbA1c levels were excluded, HNA remained significant. In model 3, which excluded the LDL-C level, systemic blood pressure, and total protein level, HNA remained an independent factor with an odds ratio of 1.12. Age and HDL-C also remained as significant factors for the prediction of multivessel CAD (Table 4).

**Table 4:** Logistic regression analysis to predict multivessel CAD.

	Model 1			Model 2			Model 3		
	OR	95% CI	*p*	OR	95% CI	*p*	OR	95% CI	*p*
HNA%	1.117	(1.002–1.246)	0.045	1.119	(1.014–1.236)	0.026	1.120	(1.020–1.229)	0.017
Age	1.083	(1.026–1.142)	0.004	1.061	(1.016–1.109)	0.008	1.048	(1.007–1.091)	0.022
HDL-C	0.940	(0.895–0.987)	0.012	0.946	(0.904–0.990)	0.017	0.959	(0.921–0.998)	0.040
LDL-C	1.011	(0.998–1.025)	0.096	1.010	(0.999–1.022)	0.080	–	–	–
SBP	1.022	(0.997–1.047)	0.090	1.015	(0.993–1.038)	0.191	–	–	–
TP	0.530	(0.228–1.232)	0.140	0.639	(0.303–1.350)	0.241	–	–	–
TnI	1.000	(1.000–1.000)	0.192	–	–	–	–	–	–
BUN	0.921	(0.813–1.045)	0.201	–	–	–	–	–	–
HbA1c	1.211	(0.877–1.671)	0.245	–	–	–	–	–	–

Numbers are reported as the odds ratio and 95% confidence interval. Model 1 was adjusted for HNA, age, HDL-C, LDL-C, SBP, TP, TnI, BUN, and HbA1c. Model 2 consisted of model 1 minus TnI, BUN, and HbA1c. Model 3 consisted of model 2 minus LDL-C, SBP, and TP.

## Discussion

This study showed that HNA was correlated with the severity of CAD independent of conventional cardiac risk factors, including age and renal function. HNA showed a moderate correlation with the Gensini score, an index of atherosclerotic disease burden that has been shown to correlate with mortality risk and cardiovascular prognosis in CAD patients, as well as associated with long-term cardiovascular prognosis in ischemic heart failure and ACS patients when used to evaluate post-coronary intervention residual coronary stenosis^20–23^. Therefore, HNA levels may also be considered as a marker of atherosclerosis in the clinical assessment of the severity of CAD.

HNA was also an independent factor for predicting multivessel CAD. Nowadays, the optimal treatment of multivessel CAD to achieve myocardial revascularization remains a challenging topic for cardiovascular specialists^24^. For patients with diabetes and severe multivessel CAD, CABG is still the standard of care based on the SYNTAX extended Survival study (SYNTAXES)^25^. Recent studies have shown that preoperative assessments using the newly developed SYNTAX score II 2020 could be useful for identifying patients with multivessel CAD who might benefit from a contemporary percutaneous coronary intervention (PCI) strategy with a favorable outcome compared with the traditional CABG strategy^26–28^. In addition, multivessel CAD was reported to be a strong risk factor for recurrent cardiovascular events in patients with stable post-myocardial infarction despite optimal medical treatment in a Danish nationwide register-based cohort study^29^. Therefore, the results of the present study suggest that HNA could be clinically relevant for easily screening multivessel CAD prior to performing invasive CAG or coronary CTA.

Previous reports have shown that HNA is associated with severe liver dysfunction and chronic kidney disease, although subjects with a medical history of liver cirrhosis, acute hepatitis and pre-existing liver dysfunction were excluded from this study. Nakatani et al. reported that age, anemia, and eGFR were significantly and independently associated with HNA in pre-dialysis CKD patients^15^. Studies reported that hemodialysis may remove uremic toxins and ameliorate the redox status of albumin by decreasing levels of HNA^30,31^. Therefore, patients who received routine dialysis or had severe renal function were also excluded from the present study. Still, our results showed that the number of coronary stenotic vessels was an independent and significant factor associated with HNA. Age and renal function were also significant in affecting the HNA level, consistent with previous reports. Additionally, our study also showed that for non-CAD individuals, HNA was significantly elevated in diabetic patients compared with that in non-diabetic patients, suggesting that poor glycemic control or hyperglycemia could lead to an elevation of oxidative stress in the circulation before the development of the overt stenosis of coronary vessels.

Elevated HNA levels have been reported to be pro-atherosclerotic. For example, high HNA levels were positively correlated with a thicker carotid intima-media thickness, and HNA itself may bind more pro-atherosclerotic lipid mediators^32,33^. Conversely, exercise as measured by the walking distance was negatively corrected with HNA levels^34^. Collectively, these reports and the present findings suggest that HNA is indeed significantly associated with the pro-atherosclerotic process.

Our study did not show the usefulness of HNA for the diagnosis of ACS. One possible reason is that the sudden plaque rupture underlying ACS is unlikely to affect HNA levels within such a short period of time. Nevertheless, elevated inflammatory parameters and hypoalbuminemia were reportedly associated with poor outcomes and higher mortality rates^35^. Therefore, further longitudinal studies may be needed to investigate the changes in HNA levels across the acute and chronic phases of myocardial infarction.

Whether interventions that lower the HNA levels reduce the risk of atherosclerotic events remains unknown. Hemodialysis, for example, has been shown to reduce HNA temporarily, although there is no evidence to suggest that this reduces the plaque burden in vessels; in contrast, one report found that HNA levels were significantly elevated in CKD patients receiving regular dialysis who had a history of cardiovascular disease (CVD) compared with those without CVD, both before and after dialysis^36^. Therefore, any interventions to lower HNA levels for the purpose of preventing CAD may need to systemically inhibit its production rather than just its intermittent removal, but further studies are needed to clarify this.

In our study, we found that HNA was negatively correlated with the total serum albumin level. This finding is supported by a previous report, in which hypoalbuminemia was found to be related to a higher risk of ischemic heart disease, heart failure, and stroke^37^. A poor nutritional status might shift the redox status of albumin, leading to a higher fraction of its oxidative form. Thus, correcting the poor nutritional status could be effective for lowering the risk of cardiovascular diseases.

The present study had several limitations. First, this study was performed at a single facility and examined a relatively small number of subjects, so larger studies are needed to validate the present findings. Second, we did not routinely evaluate plaques using intravascular ultrasound (IVUS) or optical coherence tomography (OCT) during the coronary study; it will be important to elucidate whether the type of plaques has any impact on its relationship with HNA levels and subsequent clinical outcomes. Third, we did not enroll any patients with left main complete or subtotal occlusion because of the criticality and disease severity. Finally, although the association between HNA and number of vessels with coronary stenosis was statistically significant, the wide distribution of results suggest that HNA should not be the only diagnostic tool for assessing the severity of CAD. Nevertheless, this is the first study to investigate the relationship between HNA levels and coronary stenosis as directly visualized by CAG in more than 90% of the subjects. Measurement of blood HNA is easy, highly repeatable and reproducible. Such findings could be helpful to physicians for predicting the severity of coronary stenosis and the risk of multivessel CAD in patients, especially those who are asymptomatic but have a high level of HNA.

In conclusion, the coronary plaque burden is significantly associated with a higher HNA level. This relationship is independent of renal function and age. Moreover, HNA is an independent predictor of multivessel CAD. However, HNA was not clinically significant for the diagnosis of early ACS.

## Methods

### Ethical declarations and consent to participate

The protocol of the study was approved by both the Ethics Committee of the Tokyo University Graduate School of Medicine (Approval number 10964) and the Ethics Committee of the New Tokyo Hospital (Approval number 0110-1, 0110-3). All participants or their legal guardians signed a written informed consent for both the coronary angiographic data and blood sampling data relevant to the study. All the study was performed in accordance with the Declaration of Helsinki.

### Study subjects

This cross-sectional study was performed at a single facility. A total of 140 patients who visited the emergency department or outpatient department of the New Tokyo Hospital and were suspected of having ACS or were considered to have a high risk of CAD were enrolled in this study between March 2017 and February 2018. Exclusion criteria included the presence of shock vital signs upon arrival, pregnancy, severe hepatic disorders, a history of regular hemodialysis treatment or severe renal dysfunction (creatinine > 3 mg/dL), and the regular intake of antioxidants such as vitamins C and E, since these factors have been shown to affect the measured values of the redox status of albumin. Patients with lesions involving graft stenosis at a previous coronary artery bypass graft (CABG) or in-stent restenosis after previous percutaneous coronary intervention (PCI) were also excluded from this study. Among the subjects, 113 subjects (81%) complained of chest discomfort or chest pain with or without an elevated troponin I level prior to the examinations. The remaining subjects enrolled in this study did not present with chest symptoms but exhibited objective findings such as an elevated troponin I level (n = 10), electrocardiography abnormalities (n = 4), or a new onset of an arrhythmia (n = 2); had been referred by a local hospital or clinic for detailed evaluations (n = 4); or had poorly controlled diabetes and hyperlipidemia as determined by their attending physicians (n = 7). One hundred and twenty-nine patients (92.1%) underwent CAG for direct coronary artery visualization. Nine patients (6.4%) underwent a coronary CTA, and two patients (1.4%) did not undergo any examinations because of a clear medical history of repeated episodes of coronary vasospasm without any significant coronary stenosis. Among the patients who underwent CAG, 97 patients (75%) were considered emergent and underwent the procedure on the same day as their hospital visit, and the other 32 patients (25%) received elective cardiac angiography. Before the procedures, all the patients provided informed consent and signed an agreement to participate in this study. All the documents were kept in a secure place.

### Blood sampling

Blood samples were collected from all patients upon presentation at the emergency department or the outpatient department. Routine examinations including blood urea, creatinine, liver function, albumin (Alb), total protein (TP), hemoglobin (Hb), platelets, white blood cell count (WBC), HbA1c, lipid profiles (HDL-C, LDL-C, triglyceride, total cholesterol), brain natriuretic peptide (BNP) and high sensitive C-reactive protein (hs-CRP) were performed. Myocardium-specific enzymes including creatinine kinase MB (CK-MB) and troponin I (TnI) were also taken for the early diagnosis of ACS. The estimated glomerular filtration rate (eGFR) was calculated as 194 × Cre^−1.094^ × age^−0.287^ in men, while a correlation factor of 0.739 was used for females. Patients who received elective cardiac catheterization provided a blood sample within 1 week before the examination. All the above measurements were performed in the clinical laboratory of New Tokyo Hospital. Sampling for the HNA and HMA measurements was performed using a blood collection tube filled with a stabilizer to stabilize the sample.

### Measurement of HNA and HMA

All the blood samples for HNA measurement were sent to The University of Tokyo Hospital^11,12^*.* HNA was measured using a previously reported HPLC methodology; briefly, the HPLC system consisted of an anion exchange column (50 × 7.6 mm ID) packed with a polyvinyl alcohol gel containing diethylamine. Two eluents were used: eluent A was a solution of 25 mM phosphate buffer containing 60 mM sodium sulfate (pH 6.0), and eluent B was a 250 mM magnesium chloride solution. The flow rate was 1 mL/min after equilibrating the column for 4.5 min with eluent A. We programed the linear gradient time from eluent A (100%) to eluent B (100%) for 7.5 min. The total measurement time was 12 min per sample. The sample size was 3 μL of whole blood, and the temperature was set to 40 °C. The excitation and emission wavelengths were 280 nm and 340 nm, as previously reported. The repeatability (within-day variability) and reproducibility (day-to-day variability) of the HNA measurements were both less than 1.0% (CV). The HNA and HMA levels and the total albumin area (= HNA area + HMA area) were calculated using the chromatogram peak areas obtained using HPLC. Finally, HNA% was calculated as HNA area/Total Alb area × 100.

### Diagnosis of coronary artery disease

The presence of coronary artery disease was evaluated using CAG and coronary CTA, if CAG was not available. The severity of CAD was defined according to both the number of areas with obstructive coronary artery stenosis and a score calculated by multiplying the stenotic degree by a multiplication factor for each segment (the Gensini score)^19^. Vessel stenosis was determined by the presence of significant stenosis of more than 50% at the left main trunk or 75% at the right coronary artery, left anterior descending coronary artery, or left circumflex coronary artery. Coronary artery disease was confirmed as the presence of significant stenosis in at least one coronary vessel. Left main stenosis without right coronary artery stenosis was categorized as 2-vessel disease. Multivessel coronary CAD was defined as two- or three-vessel diseases and left main stenosis. The diagnosis of ACS, including ST elevation type (STE-ACS) and non-ST elevation type (NSTE-ACS), was determined according to the guidelines of the American College of Cardiology (ACC) and the American Heart Association (AHA).

### Statistical analysis

All the analyses were performed using IBM^®^ SPSS^®^ software version 25.0 for Windows (https://www.ibm.com/support/pages/downloading-ibm-spss-statistics-25). Continuous variables were presented as the group mean ± standard deviation and categorical variables were presented as the number (%), unless otherwise described. Statistical comparisons between two continuous variables were performed using an unpaired Student *t*-test (for parametric data) or the Mann–Whitney U-test (for non-parametric data). To compare three or more groups, a one-way analysis of variations (ANOVA) was performed; if statistical significance was reached, then the Bonferroni corrections were used as a post-hoc comparison. The Pearson correlation analysis was used to investigate the Gensini score and the HNA level, as well as various clinical factors associated with HNA. A multivariate analysis was performed for the independent assessment of significant factors and myocardium-related parameters capable of predicting the HNA level. A logistic regression analysis was performed to determine the factors related to predicting multivessel CAD. A *p* value of less than 0.05 was considered statistically significant in this study. The datasets generated and analyzed in the current study are available from the corresponding authors upon reasonable request.

## Abbreviations

ACS: Acute coronary syndrome
Alb: Albumin
ANOVA: Analysis of variations
BMI: Body mass index
BNP: Brain natriuretic peptide
BUN: Blood urea nitrogen
CABG: Coronary artery bypass graft
CAD: Coronary artery disease
CAG: Coronary angiography
CK-MB: Creatinine kinase MB
CPK: Creatinine phosphokinase
Cre: Creatinine
CTA: Computed tomography angiography
CV: Coefficient of variation
DM: Diabetes mellitus
eGFR: Estimated glomerular filtration rate
Hb: Hemoglobin
HbA1c: Hemoglobin A1c
HDL-C: High-density lipoprotein cholesterol
HMA: Human mercaptalbumin
HNA: Human non-mercaptalbumin
HPLC: High performance liquid chromatography
hs-CRP: High sensitive C reactive protein
HT: Hypertension
IVUS: Intravascular ultrasound
LDL-C: Low-density lipoprotein cholesterol
NSTE-ACS: Non-ST elevation acute coronary syndrome
OCT: Optical coherence tomography
PCI: Percutaneous coronary intervention
PLT: Platelet count
SBP: Systolic blood pressure
STE-ACS: ST elevation acute coronary syndrome
TG: Triacylglycerol
TnI: Troponin I
TP: Total protein
UA: Uric acid
WBC: White blood cell
